# Supplementary figures and images for: Development of a highly specific enzyme-linked immunosorbent assay for detection of antibodies to Duck Tembusu virus using subviral particles
Source: PLoS One. 2025 Jun 27;20(6):e0326913. doi: 10.1371/journal.pone.0326913 (PMC12204544; doi:10.1371/journal.pone.0326913)

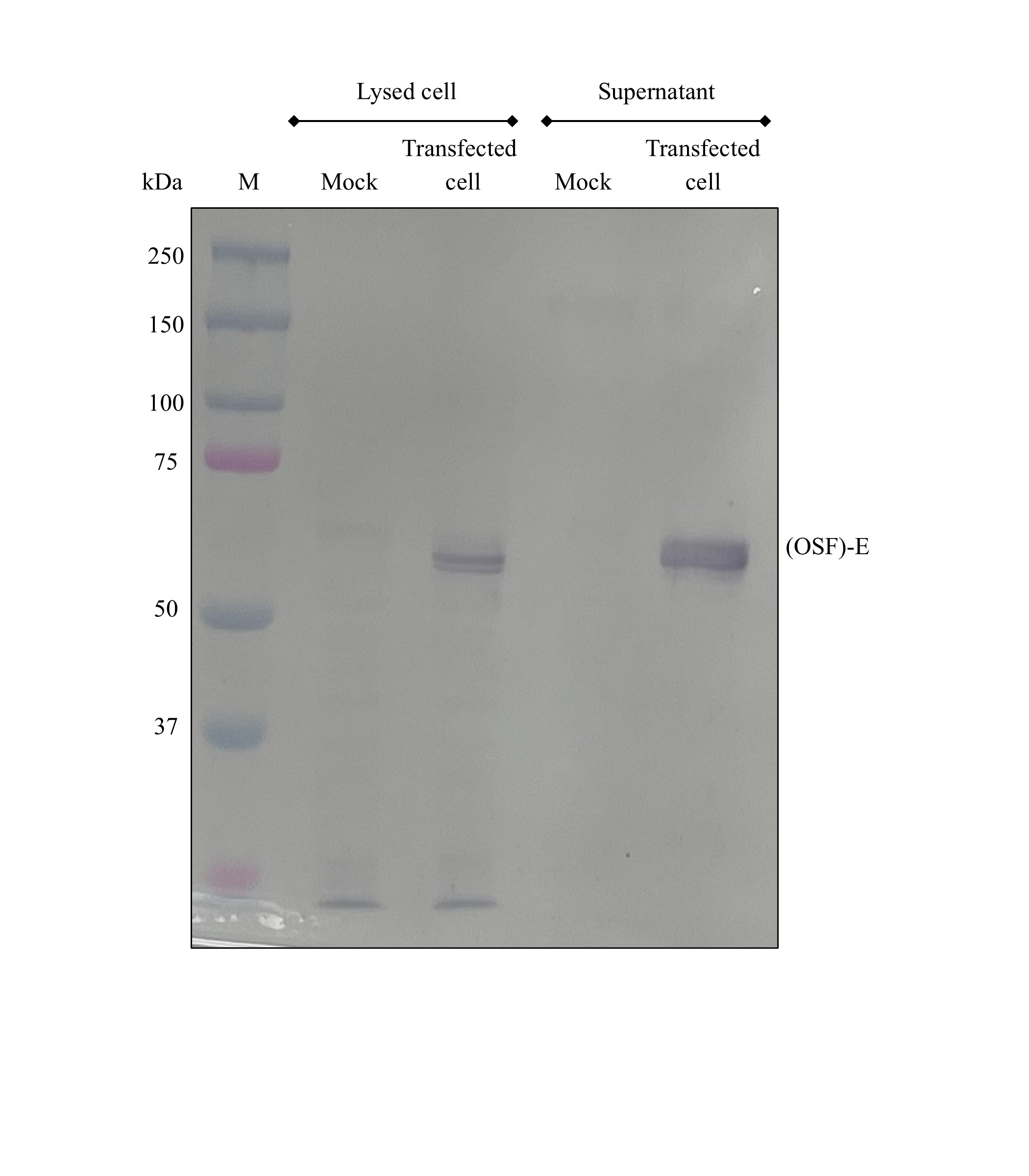

Supplement: S1 Fig — The HEK-293T cells were transfected with the recombinant plasmid or mock-transfected. Western blotting confirmed the presence of SPs in both the lysed cells and the supernatant. Lane M: Protein marker, Lysed cells: mock-transfected and transfected cell, and Supernatant: mock-transfected and transfected cell. SPs were detected using anti-Strep-Tactin® HRP (1:8,000). (TIF) [file pone.0326913.s001.tif]

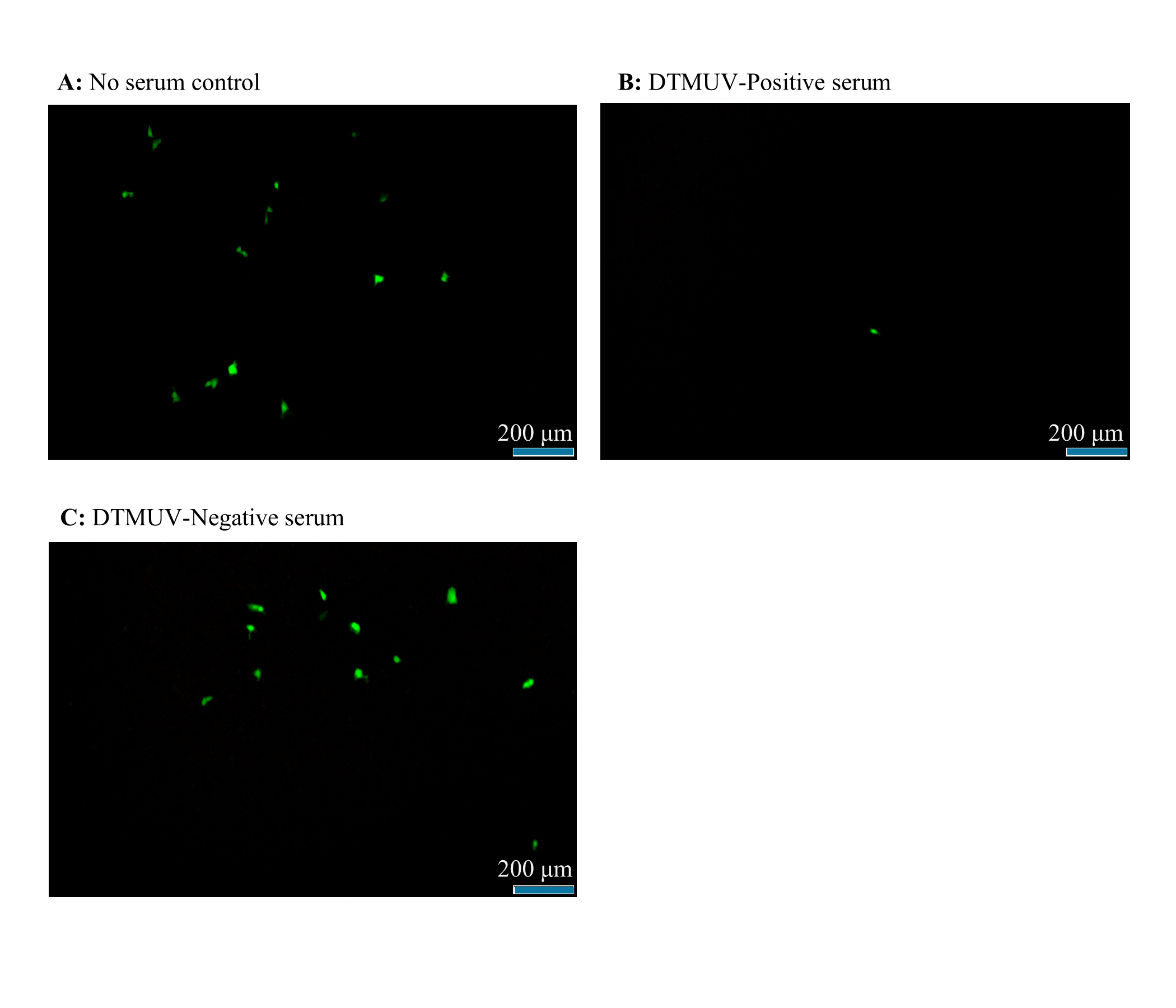

Supplement: S2 Fig — (A) No serum control, (B) DTMUV-positive serum, and (C) DTMUV-negative serum. All serum samples were diluted to a ratio of 1:5,000. Fluorescence images were captured using a fluorescence microscope at magnification of 100x. (TIF) [file pone.0326913.s002.tif]

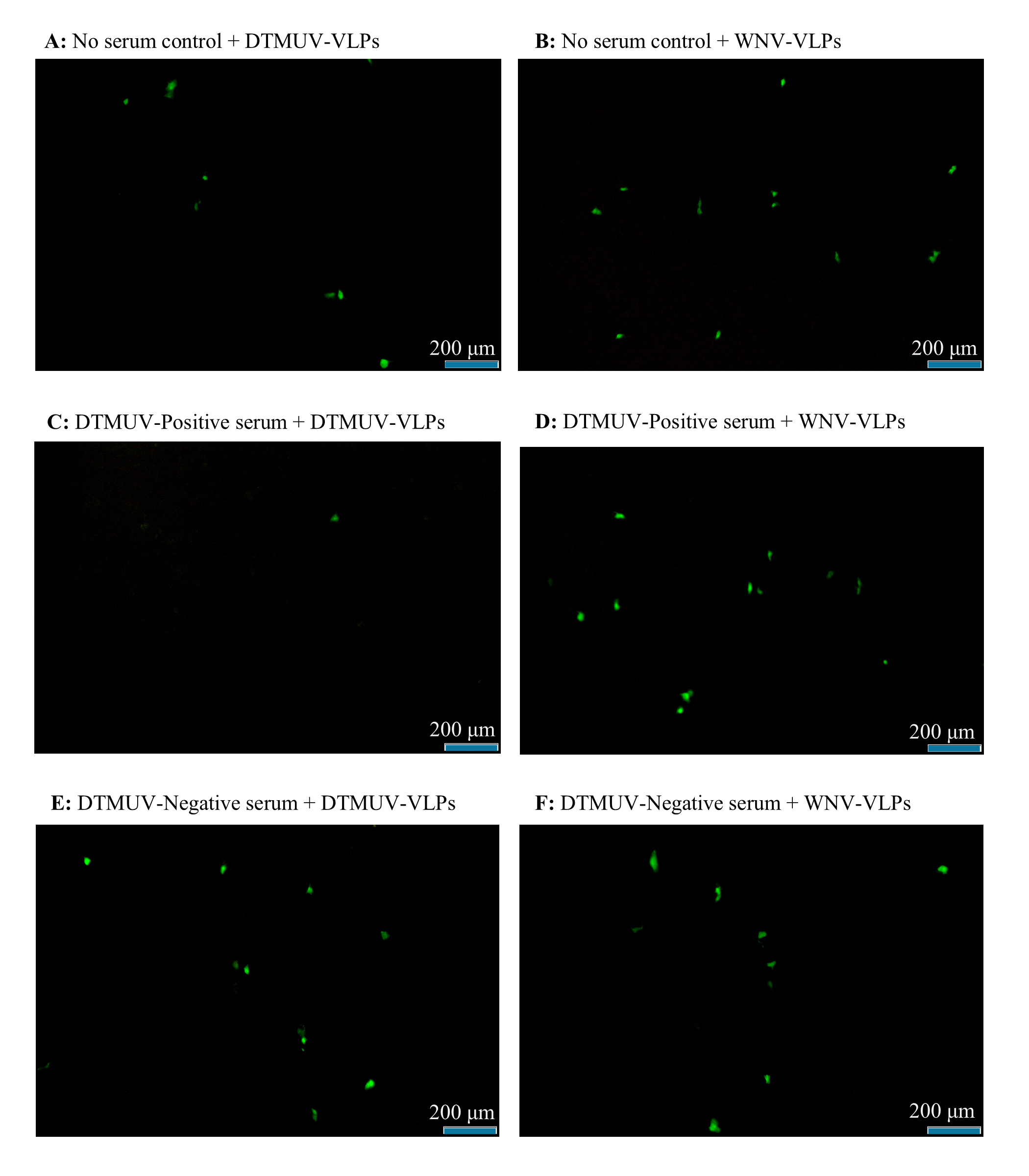

Supplement: S3 Fig — (A, B) No serum control, (C, D) DTMUV-positive serum (utilizing the same serum sample), and (E, F) DTMUV-negative serum (utilizing the same serum sample). All serum samples were diluted to a ratio of 1:5,000. Neutralization assays were performed using FRNT with either DTMUV-VLP (A, C, E) or WNV-VLP (B, D, F) to assess neutralizing activity and potential cross-reactivity. Fluorescence images were captured using a fluorescence microscope at magnification of 100 × . An 80% reduction was established as the cut-off to minimize cross-reactivity among flaviviruses and to ensure specificity in neutralization assessment. (TIF) [file pone.0326913.s003.tif]
