# Supplementary material for: Development of a highly specific enzyme-linked immunosorbent assay for detection of antibodies to Duck Tembusu virus using subviral particles
Source: PLoS One. 2025 Jun 27;20(6):e0326913. doi: 10.1371/journal.pone.0326913 (PMC12204544; doi:10.1371/journal.pone.0326913)
Supplement: S1 Table — (PDF) [file pone.0326913.s004.pdf]

S1 Table. The sensitivity and specificity of the DTMUV-SP-based ELISA compared with RT-PCR

		RT-PCR		
		Positive	Negative	
**SP-based ELISA**	Positive	17	2	19
	Negative	4	66	70
	Total	21	68	89
	**Sensitivity** (95% CI)	81.0% (57.4-93.7)		
	**Specificity** (95% CI)	97.1% (88.8-99.5)		
	**Test agreement**	0.8		

Test agreement was assessed using Kappa statistics
