# Supplementary material for: Development of a highly specific enzyme-linked immunosorbent assay for detection of antibodies to Duck Tembusu virus using subviral particles
Source: PLoS One. 2025 Jun 27;20(6):e0326913. doi: 10.1371/journal.pone.0326913 (PMC12204544; doi:10.1371/journal.pone.0326913)
Supplement: S2 Table — (PDF) [file pone.0326913.s005.pdf]

S2 Table. **Cross-reactivity test of DTMUV-SP-based ELISA-positive and negative sera to WNV-VLPs evaluated by FRNT**

**Serum NO.**	**DTMUV-FRNT (DTMUV-VLPs)**	**WNV-FRNT (WNV-VLPs)**
31	(+)	(-)
32	(+)	(-)
34	(+)	(-)
47	(+)	(-)
50	(+)	(-)
135	(+)	(-)
169	(-)	(-)
202	(-)	(-)
232	(-)	(-)
234	(-)	(-)
247	(-)	(-)
249	(-)	(-)

- Cross-reactivity testing against WNV was performed using the FRNT with WNV-VLPs, with DTMUV-VLPs as a control, on randomly selected DTMUV-SP-based ELISA-positive and negative sera. No cross-neutralization against WNV-VLPs was observed, confirming the specificity of the DTMUV-SP-based ELISA.

  (+) = Serum with a reduction in fluorescent foci exceeding 80% was considered positive;

  (–) = Serum with a reduction in fluorescent foci of less than 80% was considered negative.
