# Supplementary material for: Development of a highly specific enzyme-linked immunosorbent assay for detection of antibodies to Duck Tembusu virus using subviral particles
Source: PLoS One. 2025 Jun 27;20(6):e0326913. doi: 10.1371/journal.pone.0326913 (PMC12204544; doi:10.1371/journal.pone.0326913)
Supplement: S4 File — (PDF) [file pone.0326913.s009.pdf]

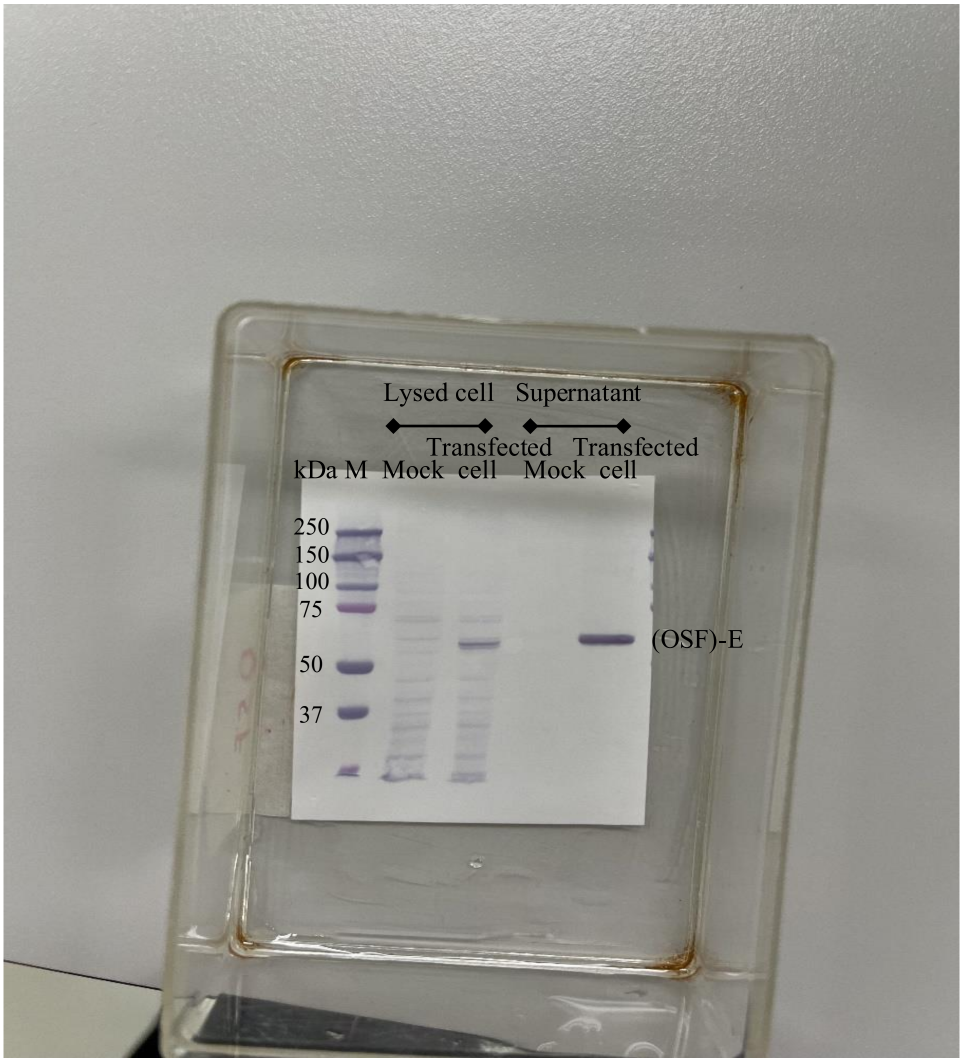

Western blot analysis confirming DTMUV-SP expression in transfected HEK-293T cells. HEK-293T cells were transfected with the recombinant plasmid encoding the SP or mock-transfected as a control. Western blotting was performed to detect the presence of SP in both the cell lysates and culture supernatants. Lane M: protein marker (kDa); Lysed cells: mock-transfected and transfected cell; Supernatant: mock-transfected and transfected cell. Detection was performed using a primary anti-DTMUV-E protein antibody (1:1,000 dilution) and a secondary anti-mouse IgG-HRP-conjugated antibody (1:1,500 dilution). A specific band corresponding to the (OSF)-E protein was observed at the expected molecular weight in both lysate and supernatant fractions of transfected cells, but not in mock controls.

These original raw images—captured using an iPhone 12 Pro Max—were used to generate the processed Western blot data presented in **Fig 1B** of the manuscript.

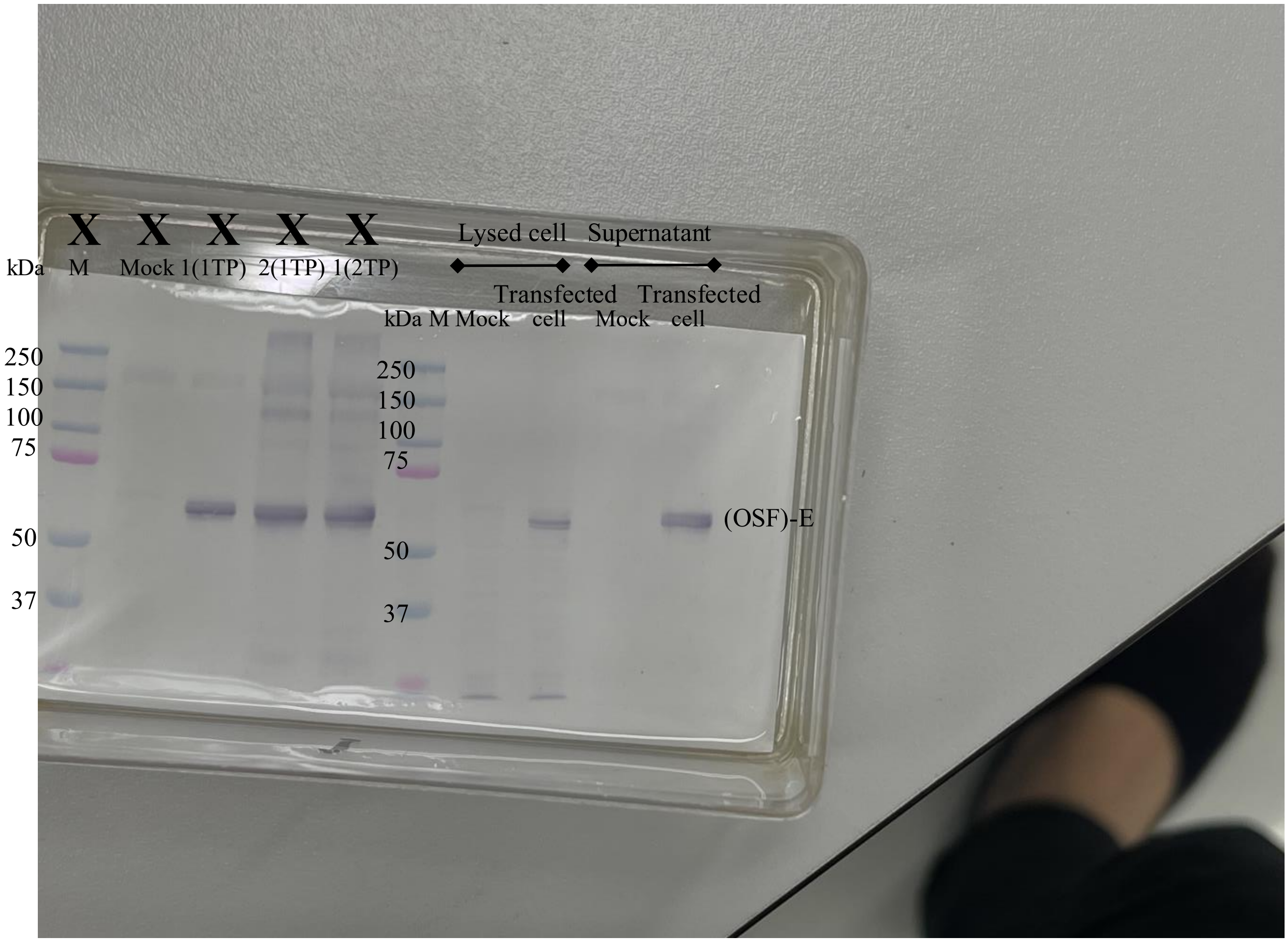

Western blot analysis confirming DTMUV-SP expression in transfected HEK-293T cells using anti-Strep-Tactin® HRP. HEK-293T cells were transfected with the recombinant plasmid encoding the SP or mock-transfected as a control. Western blotting was performed to detect the presence of SP in both the cell lysates and the culture supernatants. Lane M: protein marker; Lysed cells: mock-transfected and transfected; Supernatant: mock-transfected and transfected. Detection was performed using anti-Strep-Tactin® HRP (1:8,000 dilution). A specific band corresponding to the (OSF)-E was observed at the expected molecular weight in both lysate and supernatant fractions of transfected cells, but not in mock controls.

Portions of these original, raw blot images—captured using an iPhone 12 Pro Max—were used to generate the processed Western blot data presented in Supporting **S1 Fig.** of the manuscript.

Lanes marked with an "X" indicate unrelated experiments that were not included in the final.
